# Tau Phosphorylation in a Mouse Model of Temporal Lobe Epilepsy

**DOI:** 10.3389/fnagi.2019.00308

**Published:** 2019-11-12

**Authors:** Marianna Alves, Aidan Kenny, Gioacchino de Leo, Edward H. Beamer, Tobias Engel

**Affiliations:** Department of Physiology and Medical Physics, Royal College of Surgeons in Ireland, Dublin, Ireland

**Keywords:** tau phosphorylation, AT8, status epilepticus, epilepsy, mouse models

## Abstract

Hyperphosphorylation of the microtubule-associated protein tau and its resultant aggregation into neurofibrillary tangles (NFT) is a pathological characteristic of neurodegenerative disorders known as tauopathies. Tau is a neuronal protein involved in the stabilization of microtubule structures of the axon and the aberrant phosphorylation of tau is associated with several neurotoxic effects. The discovery of tau pathology and aggregates in the cortex of Temporal lobe epilepsy (TLE) patients has focused interest on hyperphosphorylation of tau as a potential mechanism contributing to increased states of hyperexcitability and cognitive decline. Previous studies using animal models of status epilepticus and tissue from patients with TLE have shown increased tau phosphorylation in the brain following acute seizures and during epilepsy, with tau phosphorylation correlating with cognitive deficits in patients. Suggesting a functional role of tau during epilepsy, studies in tau-deficient and tau-overexpressing mice have demonstrated a causal role of tau during seizure generation. Previous studies, analyzing the impact of seizures on tau hyperphosphorylation, have mainly used animal models of acute seizures. These models, however, do not replicate all aspects of chronic epilepsy. In this study, we investigated the effects of acute seizures (status epilepticus) and chronic epilepsy upon the expression and phosphorylation of tau using the intra-amygdala kainic acid (KA)-induced status epilepticus mouse model. Status epilepticus resulted in an immediate increase in total tau levels in the hippocampus, in particular, the dentate gyrus, and phosphorylation of the AT8 epitope (Ser202, Thr205), with phosphorylated tau mainly localizing to the mossy fibers of the dentate gyrus. During epilepsy, abnormal phosphorylation of tau was detected again at the AT8 epitope with lower total tau levels in the CA3 and CA1 subfields of the hippocampus. Chronic epilepsy in mice also resulted in a strong localization of AT8 phospho-tau to microglia, indicating a distinct pattern of tau hyperphosphorylation during chronic epilepsy compared to status epilepticus. Our results reaffirm previous observations of tau phosphorylation post-status epilepticus, but also elaborate on tau alterations in epileptic mice which more faithfully mimic TLE. Our results confirm seizures affect tau hyperphosphorylation, however, suggest epitope-specific phosphorylation of tau and differences in cell-specific localization according to disease progression.

## Introduction

Tauopathies are a diverse group of neurodegenerative disorders characterized by misfolded, aggregated and aberrant forms of the microtubule-associated protein tau (Götz et al., 2019). These tauopathies have been traditionally limited to diseases in which tau was a primary pathology feature such as Alzheimer’s Disease, Pick’s Disease, progressive supranuclear palsy and chronic traumatic encephalopathy (Götz et al., 2019). Recent studies are identifying, however, an increasing number of disorders with tau pathology. These new tau-associated disorders include the neurodegenerative disorders Huntington’s and Parkinson’s Disease as well as Temporal lobe epilepsy (TLE; Fernández-Nogales et al., 2014; Tai et al., 2016, p. 4; Cisbani et al., 2017, p. 3). TLE is the most common form of acquired epilepsy in adults and particularly prone to pharmacoresistance (Schmidt and Löscher, 2005) and, although not defined as a neurodegenerative disease, tau-based aggregates which directly correlate with cognitive decline have been identified in TLE patient brain (Thom et al., 2011; Tai et al., 2016, p. 4).

Tau is a microtubule-associated protein, primarily found in neurons and located in axons (Janning et al., 2014). Although tau can be targeted by numerous kinases *in vivo*, this is limited to a small group of well-characterized kinases which includes Glycogen synthase kinase-3β (GSK-3β), Cyclin-dependent kinase 5 (CDK5) and Microtubule-affinity-regulating kinase (MARK; Martin et al., 2013b). These kinases work in tandem with a group of phosphatases [Protein phosphatase-1, -2 and -5 (PP1/2/5)] to curate the modifications and thereby the function of tau in the cell (Martin et al., 2013a). This cycle of phosphorylation is ultimately the mechanism by which abnormal forms of tau are typically generated with imbalances in kinase/phosphatase activity resulting in hyperphosphorylation of tau, leading to misfolding and aggregation (Götz et al., 2019). The primary mechanism by which these aberrant modifications induce neurodegeneration in cells is heavily debated and can be loosely divided into two groups including a loss-of-function, where hyperphosphorylation of tau blocks its interaction and stabilization of microtubule structures possibly impacting on axonal transport and synaptic transmission (Feinstein and Wilson, 2005), or a toxic-gain-of function where hyperphosphorylation of tau drives aberrant interactions such as disruption of the trafficking and anchoring of receptors in dendritic compartments or negatively affecting mitochondrial function (Hoover et al., 2010; Lasagna-Reeves et al., 2011).

Tau is well known to have a significant influence on neuronal activity and seizures. Several studies have shown tau to be integral to hyperactivity in mouse models of status epilepticus where knock-down of tau is neuroprotective and ameliorates seizure activity (Holth et al., 2013), while tau overexpression models have been shown to result in hyperexcitability and seizures (García-Cabrero et al., 2013). These findings are, however, at odds with conflicting studies where tau hyperphosphorylation and overexpression have been shown to dampen neuronal excitability (Angulo et al., 2017; Busche et al., 2019). The mechanism behind this bi-directional effect in which both aberrant increases and decreases in tau induce a synaptic dampening and reduction in excitatory activity remains unexplained and requires further investigation of the underlying molecular processes.

Studies in experimental seizure models have identified a wave of increased kinase activity in neurons resulting from seizure activity which includes several kinases which all putatively act on tau phosphorylation: GSK-3β, CDK5, extracellular signal-regulated kinase (ERK) and protein kinase A (PKA; Liang et al., 2009; Gangarossa et al., 2015). The induction of seizures in mice has also been shown to cause long-term downregulation of the phosphatase PP2A, potentially compounding the imbalance of tau hyperphosphorylation (Liang et al., 2009). Previous investigations of seizure-induced changes in tau phosphorylation have been limited to the induction of convulsive status epilepticus *via* a systemic injection of kainic acid (KA) which produces inconsistent pathology and hippocampal epileptogenesis (Sloviter et al., 2007). The processes and cellular responses of the periodic and less severe seizure activity in chronic epilepsy are, however, distinct from the initial intense excitation of status epilepticus.

In this present study, we investigated the impact of status epilepticus and chronic epilepsy on the phosphorylation and localization of tau using the intra-amygdala KA mouse model of status epilepticus (Mouri et al., 2008). The intra-amygdala KA mouse model is a refined model for the focalized induction of status epilepticus and reliable stimulation of epileptogenesis (Henshall et al., 2000; Mouri et al., 2008) and, therefore, the study of tau phosphorylation in this model may help to shed further light on tau pathology seen in TLE patients.

## Materials and Methods

### Animals Model of Status Epilepticus

All animal experiments were performed in accordance with the principles of the European Communities Council Directive (2010/63/EU). Procedures were reviewed and approved by the Research Ethics Committee of the Royal College of Surgeons in Ireland (REC 1322) and the Irish Health Products Regulatory Authority (AE19127/P038). All efforts were maximized to reduce the number of animals used in this study. Mice used in our experiments were 8- to 12-week-old male C57BL/6 mice, obtained from the Biomedical Research Facility, Royal College of Surgeons in Ireland (Dublin, Ireland). Animals were housed in a controlled biomedical facility on a 12 h light/dark cycle at 22 ± 1°C and humidity of 40–60% with food and water provided *ad libitum*. Status epilepticus was induced by a unilateral stereotaxic microinjection of KA (Sigma-Aldrich, Arklow, Ireland) into the amygdala, as described (Brennan et al., 2013). A guide cannula was affixed over the dura (coordinates from Bregma: AP = −0.94; L = −2.85 mm) and the entire skull assembly fixed in place with dental cement. Then a 31-gauge internal cannula was inserted into the lumen of the guide cannula to inject KA into the amygdala [0.3 μg in 0.2 μl vehicle; phosphate-buffered saline (PBS), pH adjusted to 7.4] at a flow rate of 0.02 μl per second over 10 s. Non-seizure control mice received an intra-amygdala injection of 0.2 μl of sterile PBS. Mice typically develop status epilepticus shortly following intra-amygdala KA injection undergoing typical behavioral changes scored according to a modified Racine Scale. This includes: Score 1, immobility and freezing; Score 2, forelimb and or tail extension, rigid posture; Score 3, repetitive movements, head bobbing; Score 4, rearing and falling; Score 5, continuous rearing and falling; Score 6, severe tonic–clonic seizures (Jimenez-Mateos et al., 2012). A single dose of the anticonvulsant lorazepam (8 mg/kg, intraperitoneal) was administered 40 min after KA to curtail status epilepticus and reduce mortality and morbidity. Mice were euthanized at different time-points (1 h, 4 h, 8 h, 24 h and 14 days) after anticonvulsant administration.

### Microdissection of Hippocampal Subfields

To analyze the different hippocampal subfields separately, mouse brains were removed using sharp laboratory scissors and placed into a petri dish on top of a cold board. Following the separation of the cerebellum, the two brain hemispheres were separated. Then, using a dissecting microscope, the whole hippocampus was separated from the cortex. Following the identification of the boundaries between the dentate gyrus, cornus ammonis (CA)1 and CA3, the three hippocampal subfields were separated and immediately put on dry ice and stored at −80°C.

### Drug Treatment

The GSK-3 inhibitor NP031112 [Tideglusib, NP12 (Domínguez et al., 2012); Sigma-Aldrich, Arklow, Ireland] was administered with a 2 μl infusion of intra-cerebro-ventricular (i.c.v.) Dimethyl sulfoxide (DMSO) 15 min after the administration of the anticonvulsant lorazepam into the ventricle (ventricle volume was calculated as 30 μl) to reach a final concentration of 100 μM (Engel et al., 2018). In the vehicle group, animals were injected with 2 μl of sterile DMSO.

### Western Blotting

Western blotting was performed as described previously (Engel et al., 2017). Following quantification of protein concentration, 30 μg of protein samples were boiled in gel-loading buffer and separated by sodium dodecyl sulfate–polyacrylamide gel electrophoresis. Proteins were transferred to nitrocellulose membranes and probed with the following primary antibodies: Tau-1 (1:1,000, Merck Millipore, Arklow, Ireland), AT8 (1:100, Invitrogen, CA, USA), PHF-1 (1:1,000, Abcam, Cambridge, UK) and GAPDH (1:1,000, Cell Signalling, Dublin, Ireland). Next, membranes were incubated with horseradish peroxidase-conjugated goat anti-rabbit or anti-mouse secondary antibodies (Isis Limited, Bray, Ireland). Protein bands were visualized using Fujifilm LAS-4000 system (Fujifilm, Tokyo, Japan) with chemiluminescence.

### RNA Extraction and Real-Time Quantitative Polymerase Chain Reaction

RNA extraction was undertaken as previously described using TRIzol^®^ (QIAzol Lysis Reagent, Qiagen, Hilden, Germany; Jimenez-Mateos et al., 2015). Five-hundred microgram of total RNA was used to generate complementary DNA by reverse transcription using SuperScript^®^ III reverse transcriptase enzyme (Thermo Fisher Scientific, Waltham, MA, USA). Quantitative real-time PCR was performed using a LightCycler 1.5 (Roche Diagnostics GmbH, Mannheim, Germany) in combination with QuantiTect^®^ SYBR^®^ Green PCR Kit (Qiagen, Hilden, Germany) as per the manufacturer’s protocol, and 1.25 μM of primer pair was used. Data were analyzed by LightCycler 1.5 software and normalized to the expression of *β-actin* and represented as relative quantification values. Primers were designed using Primer3 software^1^. Primer sequences: *tau* F: aatcagtctccacaccccag, R: actacaacgtaacagggcga; *tau-3R* F: cccagc tctggtgaacctcca, R: tcacaaaccctgcttggccagand and *β-actin*, F: gggtgtgatggtgggaatgg, R: ggttggccttagggttcagg.

### Fluoro-Jade B Staining

Fluoro-Jade B (FjB) staining was carried out as before (Engel et al., 2013). Twelve micrometer coronal sections at the medial level of the hippocampus (Bregma AP = −1.94 mm) were cut on a cryostat. Tissue was fixed in formalin, rehydrated in ethanol, and then transferred to a 0.006% potassium permanganate solution followed by incubation with 0.001% FjB (Chemicon Europe Limited, Chandlers Ford, UK). The sections were mounted in DPX mounting solution. Using an epifluorescence microscope, FjB-positive cells within the ipsilateral hippocampus including all subfields (dentate gyrus, CA1 and CA3 regions) were counted by a person unaware of treatment under a 40× lens in two adjacent sections and the average determined for each animal.

### 3,3′-Diaminobenzidine Immunohistochemistry

Mice were transcardially perfused with 4% paraformaldehyde (PFA) and postfixed for an additional 24 h. Brains were then transferred to PBS and immersed into 4% agarose before sectioning. 3,3′-Diaminobenzidine (DAB) staining was carried out as before (Engel et al., 2006). Slices were treated with 1% H_2_O_2_ for 45 min to inactivate endogenous peroxidases. This was followed by an incubation with blocking solution (10 ml: 8.3 ml 1× PBS, 1 ml 10% BSA, 0.5 ml 5% FBS, 0.2 ml Triton-X-100) for 1 h and primary antibody (AT8, 1:100, Invitrogen, CA, USA) overnight at 4°C. On the following day, tissue sections were washed 3× with PBS for 10 min each and incubated with Vectastain kit (Vector Laboratories, Burlingame, CA, USA; one drop of biotinylated antibody and three drops of horse/donkey serum were mixed in 10 ml of 1% BSA-PBS). This solution was added to the tissue and incubated for 90 min at room temperature. Three washes were performed with PBS for 10 min followed by a 90 min incubation with Avidin (ABC) peroxidase complex at room temperature (two drops of reagent A and reagent B in 10 ml of 1% BSA-PBS). Slices were washed with PBS for 20 min and immersed in DAB solution (Sigma-Aldrich, Arklow, Ireland) for approximately 10 min. Then, slices were mounted using FluorSave^TM^. Staining was examined under a light microscope.

### Immunofluorescence

As for DAB staining, mice were transcardially perfused with 4% PFA and postfixed for an additional 24 h. Following immersed into 4% agarose, brains were sectioned into 30 μm thick sagittal sections using the VT1000S vibratome (Leica Microsystems, Wetzlar, Germany). Tissue sections were incubated with 0.1% Triton/PBS and 1 M glycine, followed by blocking with 1% BSA in PBS for 45 min. The sections were then incubated with the primary antibody AT8 (1:100, Invitrogen, CA, USA) overnight. This was followed by washing with PBS and a second incubation for 2 h at room temperature with a second primary antibody against NeuN (1:400, Millipore, Arklow, Ireland), GFAP (1:400, Sigma-Aldrich, Arklow, Ireland), Iba-1 (1:400, Wako, Fuggerstrasse, Germany) or Zinc Transporter 3 (ZnT3; 1:200, Synaptic Systems GmbH, Goettingen, Germany). After washing in PBS, tissue was incubated with fluorescent secondary antibodies [AlexaFluor-488 or AlexaFluor-568 (BioSciences, Dublin, Ireland)] followed by a short incubation with DAPI. FluorSave^TM^ (Millipore, Arklow, Ireland) was used to cover the tissue, and confocal images were taken with a TCR 6500 microscope (Leica Microsystems, Wetzlar, Germany) equipped with four laser lines (405, 488, 561, and 653 nm) using a 40× immersion oil objective (NA 1.3; Leica Microsystems).

### Data Analysis

Data were analyzed for statistical significance using StatView. Parametric analysis was carried out using Student’s *t*-test and one-way ANOVA with Fishers *post hoc* multiple comparisons analysis. Data are presented as mean ± standard error of the mean (SEM).

## Results

### Increased Tau Phosphorylation in the Hippocampus Following Intra-Amygdala Kainic Acid-Induced Status Epilepticus

**Figure 1:**
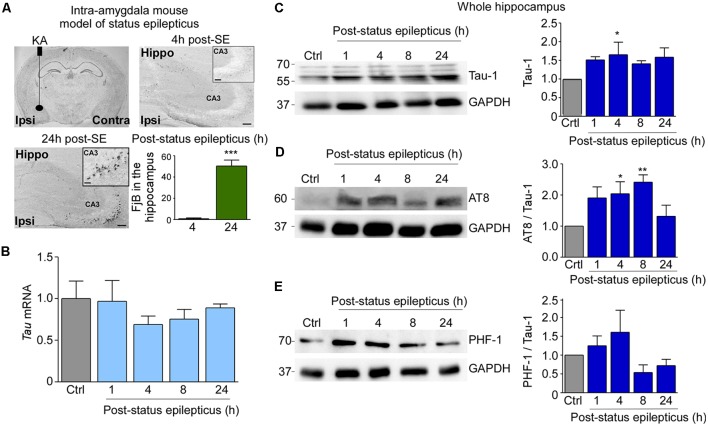
Tau protein expression and tau phosphorylation in the hippocampus post-status epilepticus. **(A)** Illustration of intra-amygdala injection of Kainic acid (KA) in mice for the induction of status epilepticus and chronic epilepsy. Flouro-jade B (FjB) staining shows neurodegeneration in the ipsilateral hippocampus at 24 h post-KA injection. Note: absence of FjB-positive cells at 4 h post status epilepticus (*n* = 4 per group). FjB-positive cells are mainly localized to the ipsilateral CA3 subfield of the hippocampus. Scale bar = 200 μm. **(B)** *Tau* mRNA levels quantified by RT-qPCR showing no changes between control and post-status epilepticus (*n* = 4 per group). **(C–E)** Representative Western blot (*n* = 1/lane) and corresponding graphs showing an increase in total tau (Tau-1; Ctrl vs. 4 h, *p* = 0.03), AT8/Tau-1 (Ctrl vs. 4 h, *p* = 0.03; Ctrl vs. 8 h, *p* = 0.005) and PHF-1/Tau-1 protein levels in the ipsilateral hippocampus post-status epilepticus. GAPDH is shown as a loading control (*n* = 4 per group). One-way ANOVA with Fisher’s *post hoc* test. Data are presented as mean ± standard error of the mean (SEM). *p < 0.05, **p < 0.01, ***p < 0.001.

To study the effects of status epilepticus (prolonged, damaging seizure) on tau expression and phosphorylation in the hippocampus *in vivo*, status epilepticus was induced in C57Bl/6 wild-type mice by an intra-amygdala injection of KA (Engel et al., 2013). In this model, status epilepticus leads to a distinct cell death pattern in the brain which is mainly restricted to the ipsilateral cortex and hippocampus. Within the ipsilateral hippocampus, the CA3 subfield is the most affected while the dentate gyrus and CA1 are mainly spared from cell death (Mouri et al., 2008; Figure 1A). All mice treated with intra-amygdala KA develop epilepsy following a short latency period of 3–5 days experiencing 2–5 spontaneous seizures per day (Mouri et al., 2008).

As a first approach, we analyzed whether status epilepticus has an effect on the expression of tau in the ipsilateral hippocampus. RT-qPCR revealed hippocampal *tau* mRNA levels to be relatively unchanged compared to control at all time-points analyzed post-status epilepticus (1 h–24 h; Figure 1B). Moreover, no induction of the *3R tau* isoform was observed in the hippocampus following status epilepticus (Supplementary Figure S1A). In contrast to *tau* mRNA levels, hippocampal tau protein levels detected *via* the Tau-1 antibody were increased at 4 h post-status epilepticus and remained elevated for up to 24 h [Ctrl vs. 4 h, 1.65 ± 0.3373 (mean ± SEM), *p* = 0.03; ANOVA *post hoc* Fisher’s test; Figure 1C]. Next, changes in the phosphorylation of tau in the hippocampus were analyzed using two antibodies recognizing different phosphorylation sites within the tau protein. This included AT8 (phosphorylation of Ser202/Thr205; Goedert et al., 1995) and PHF-1 (phosphorylation of Ser396/Ser404; Otvos et al., 1994). Western blotting of hippocampal samples revealed phosphorylation at the AT8 epitope was significantly increased at 4 h and 8 h post-status epilepticus when normalized to total tau levels [Ctrl vs. 4 h, 2.041 ± 0.384 (mean ± SEM), *p* = 0.03; Ctrl vs. 8 h, 2.413 ± 0.2357 (mean ± SEM), *p* = 0.005; ANOVA *post hoc* Fisher’s test] with this increase tapering off at 24 h post-status epilepticus (Figure 1D). Increases in tau phosphorylation at the AT8 epitope were also present at 1 h, 4 h and 8 h when not corrected to total tau levels [Ctrl vs. 1 h post-status epilepticus: 2.954 ± 0.6304 (mean ± SEM), *p* = 0.01; Ctrl vs. 4 h post-status epilepticus: 3.111 ± 0.3991 (mean ± SEM), *p* = 0.008; Ctrl vs. 8 h: 3.401 ± 0.4129 (mean ± SEM), *p* = 0.003; ANOVA *post hoc* Fisher’s test; Supplementary Figure S1B]. Western blots using the phospho-tau antibody PHF-1 revealed no significant increase when normalized to total tau levels (Figure 1E). PHF-1 was, however, increased at 4 h post-status epilepticus when not corrected to Tau-1 [Ctrl vs. 4 h: 2.261 ± 0.6531 (mean ± SEM), *p* = 0.04; ANOVA with Fisher’s *post hoc* test; Supplementary Figure S1B].

Thus, intra-amygdala KA-induced status epilepticus leads to an increase in total tau levels and in tau phosphorylation in the hippocampus.

### Subfield-Specific Tau Phosphorylation in the Hippocampus Post-Status Epilepticus

With AT8 showing the strongest increase in tau phosphorylation post-status epilepticus, we next sought to identify the localization of AT8 phospho-tau following status epilepticus using immunological staining of hippocampal tissue sections.

**Figure 2:**
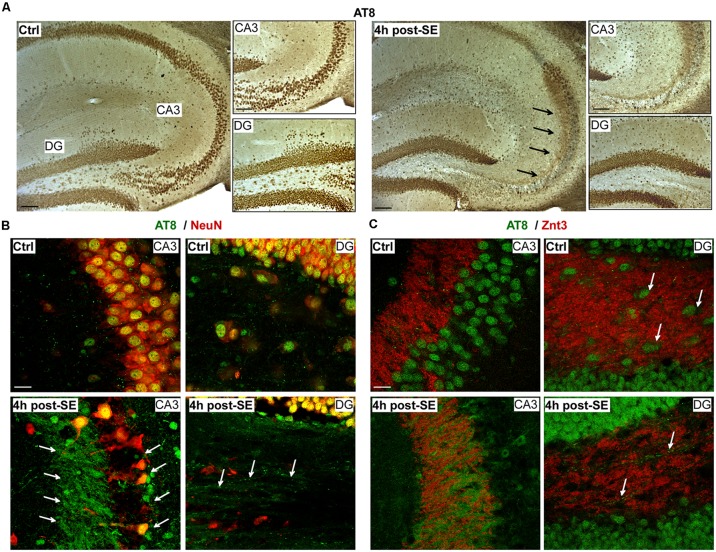
Localization of tau phosphorylation in the hippocampus following status epilepticus. **(A)** Representative 3,3′-Diaminobenzidine (DAB) staining (from a total of three different mice) showing loss of AT8 phospho-tau in the stratum pyramidal and lucidum region (CA3). In contrast, AT8-positive tau was increased in mossy fibers 4 h post-status epilepticus. Arrows indicate AT8 phospho-tau localization to mossy fibers. Scale bar = 50 μm. **(B,C)** Representative photomicrographs (from a total of three different mice) showing AT8-positive phospho-tau co-localized with the neuronal marker NeuN under physiological conditions and 4 h post-status epilepticus. Co-localization of AT8 with the Zinc Transporter 3 (Znt-3) confirmed the presence of AT8-positive phospho-tau on mossy fibers in the CA3 subfield and the hilus. Of note, decreased AT8 immunoreactivity in hilar interneurons post-status epilepticus (green = AT8, red = NeuN or Znt-3). Scale bar = 25 μm.

DAB immunostaining for AT8 4 h post-status epilepticus revealed a loss of phosphorylated tau within the stratum pyramidal and lucidum region of CA3 and an apparent increase in the mossy fibers (Figure 2A). Loss of AT8 immunoreactivity in the CA3 subfield was confirmed using co-immunostainings with the neuronal marker NeuN. Double immunofluorescence using antibodies recognizing AT8 phospho-tau and the synaptic and mossy fibers localized protein Zinc Transporter 3 (Znt3; Wenzel et al., 1997) confirmed co-localization of AT8 with mossy fibers (Figures 2B,C). Interestingly, AT8 immunoreactivity of hilar interneurons seemed to disappear post-status epilepticus (Figure 2C).

**Figure 3:**
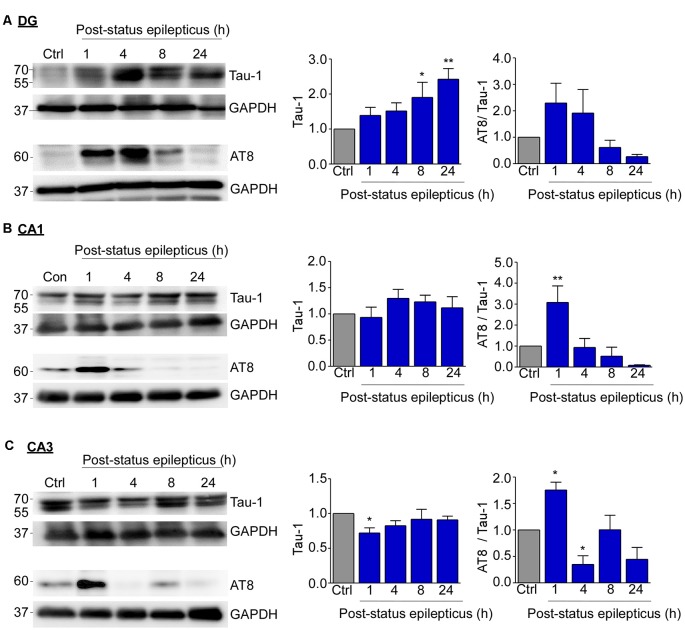
Subfield-specific tau phosphorylation in the hippocampus following status epilepticus. **(A)** Representative Western blots (*n* = 1/lane) and corresponding graphs showing an increase in total tau levels at 8 h and 24 h post-status epilepticus in the dentate gyrus (Tau-1: Ctrl vs. 8 h, *p* = 0.045; Ctrl vs. 24 h, *p* = 0.005). No significant changes were found in AT8 phosphorylation in the dentate gyrus post-status epilepticus when normalized to Tau-1. **(B)** No changes in tau expression occurred in CA1 and, in CA3 **(C)**, tau levels were found to be slightly decreased 1 h following status epilepticus (Ctrl vs. 8 h, *p* = 0.037). When normalized to total tau levels, AT8 phosphorylated tau protein levels were increase at 1 h post-status epilepticus in the CA1 (AT8/Tau: Ctrl vs. 1 h, *p* = 0.008) and CA3 (AT8/Tau: Ctrl vs. 1 h, *p* = 0.016; Ctrl vs. 4 h, *p* = 0.033) hippocampal subfields. One-way ANOVA with Fisher’s *post hoc* test. Data are presented as mean ± SEM. *p < 0.05, **p < 0.01.

Next, we analyzed tau expression and phosphorylation post-status epilepticus within each hippocampal subfield (dentate gyrus, CA1 and CA3) separately *via* Western blotting. This revealed that total tau levels were increased at 8 h and 24 h in the dentate gyrus following status epilepticus [Ctrl vs. 8 h, 1.904 ± 0.4338 (mean ± SEM), *p* = 0.045; Ctrl vs. 24 h, 2.424 ± 0.3041 (mean ± SEM), *p* = 0.005; ANOVA *post hoc* Fischer’s test; Figure 3A]. No changes occurred in CA1 (Figure 3B). In CA3, tau levels were found to be slightly decreased shortly following status epilepticus at 1 h [Ctrl vs. 1 h, 0.7211 ± 0.0731 (mean ± SEM), *p* = 0.037; ANOVA *post hoc* Fisher’s test; Figure 3C]. When normalized to total tau levels, the only statistically significant increase in tau phosphorylation on the AT8 epitope was at 1 h post-status epilepticus in the CA1 and CA3 hippocampal subfields, while no significant changes were present in the dentate gyrus [CA1 Ctrl vs. 1 h, 3.1 ± 0.79 (mean ± SEM), *p* = 0.008; CA3 Ctrl vs. 1 h, 1.756 ± 0.1471 (mean ± SEM), *p* = 0.016; ANOVA *post hoc* Fisher’s test; Figures 3A–C]. AT8-dependent tau phosphorylation levels seemed then to progressively decreased in all subfields at each time point thereafter reaching significance at 4 h post-status epilepticus in the CA3 subfield [Ctrl vs. 4 h post-status epilepticus, 0.3496 ± 0.1625 (mean ± SEM), *p* = 0.033; ANOVA *post hoc* Fisher’s test; Figures 3A–C]. A similar trend was observed when normalizing to the loading control GAPDH (Supplementary Figure S1C).

We have previously identified a functional involvement for GSK-3β, one of the main kinases targeting tau, during seizure generation and seizure-induced cell death in the intra-amygdala KA mouse model (Engel et al., 2018). To test whether GSK-3β contributes to tau phosphorylation during status epilepticus, mice were pre-treated with the GSK-3 inhibitor NP12 prior to the induction of status epilepticus. The inhibition of GSK-3 had no significant effect on the level of phosphorylation at the AT8 and PHF-1 epitope in the whole hippocampus indicating that GSK-3β alone may not be the primary driver of phosphorylation of tau during status epilepticus (Supplementary Figure S2A). In addition, the effect of the GSK-3 inhibitor NP12 was examined in the CA3 subfield of the hippocampus. Again, no changes in tau-phosphorylation could be observed (Supplementary Figure S2B).

In summary, tau expression and tau phosphorylation increases following intra-amygdala KA-induced status epilepticus in the hippocampus, with tau expression changes most evident in the dentate gyrus and CA3 and tau phosphorylation most evident in the CA1 and CA3 subfields.

### Decreased Tau Levels and Increased Tau Phosphorylation in the Hippocampus of Mice During Epilepsy

**Figure 4:**
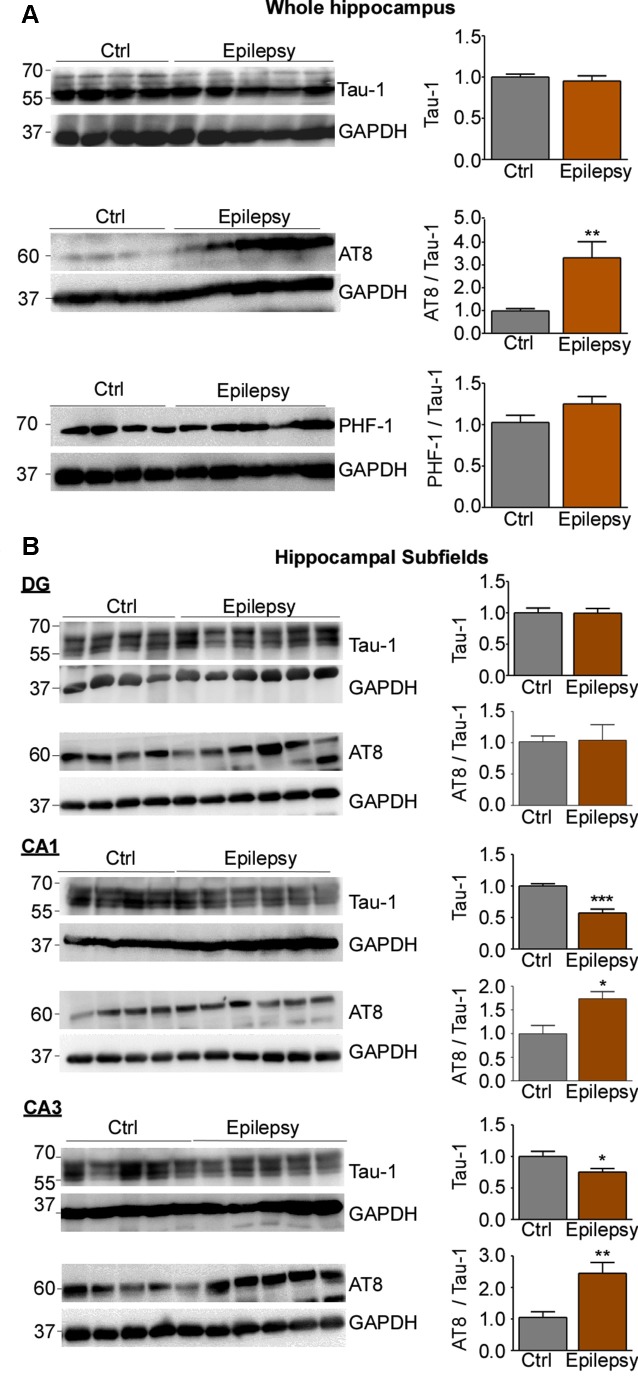
Expression and phosphorylation of tau in the hippocampus of epileptic mice. **(A)** No change in the total tau protein levels in the ipsilateral hippocampus was detected between control and epileptic mice. While AT8 phospho-tau was significantly increased in the hippocampus of epileptic mice (*p* = 0.004), no change was observed for PHF-1-positive phospho-tau. **(B)** Representative Western blots and corresponding graphs showing no obvious change in tau expression in the dentate gyrus of epileptic mice. Tau levels were, however, significantly decreased in CA1 and CA3 in epileptic mice (CA1: Ctrl vs. Epilepsy, *p* = 0.0008; CA3: Ctrl vs. Epilepsy, *p* = 0.027). When normalized to total tau, tau phosphorylation in AT8 was increased in CA1 and CA3, while no changes were observed in the dentate gyrus (CA1: Ctrl vs. Epilepsy, *p* = 0.014; CA3: Ctrl vs. Epilepsy, *p* = 0.0073). GAPDH is shown as a loading control [CA1: *n* = 4 (Ctrl) and *n* = 6 (Epilepsy); CA3 and DG: *n* = 5 (Ctrl) and *n* = 6 (Epilepsy), unpaired Student’s *t*-test. Data are presented as mean ± SEM]. *p < 0.05, **p < 0.01, ***p < 0.001.

To test whether tau phosphorylation is also increased during epilepsy, hippocampal tissue was analyzed using the same tau phosphorylation-recognizing antibodies as before in brain tissue of mice killed at 14 days post-intra-amygdala KA-induced status epilepticus, time-point when all mice have usually experienced at least 1 week of chronic epilepsy (Mouri et al., 2008; Jimenez-Mateos et al., 2012; Engel et al., 2013; Jimenez-Pacheco et al., 2016). Western blotting using whole hippocampal extracts revealed, in contrast to our findings during status epilepticus, no changes in total tau expression compared to controls (Figure 4A). While AT8 phospho-tau was significantly increased in the hippocampus of epileptic mice [Ctrl vs. Epi, 3.315 ± 0.7054 (mean ± SEM), *p* = 0.004; *t*-test], PHF-1-positive phospho-tau showed no changes (Figure 4A). Tau phosphorylation at the AT8 epitope was also significantly higher in epileptic mice without correction for total tau [Ctrl vs. Epi, 2.860 ± 0.4957 (mean ± SEM), *p* = 0.002; *t*-test], while PHF-1 showed no significant changes (Supplementary Figure S3A).

As before for status epilepticus, we then analyzed tau expression and phosphorylation for each hippocampal subfield separately. While there was no obvious change in tau expression in the dentate gyrus, tau levels were significantly decreased in CA1 and CA3 [Ctrl vs. Epi: CA1, 0.5713 ± 0.06162 (mean ± SEM), *p* = 0.0008; CA3, 0.7537 ± 0.05271 (mean ± SEM), *p* = 0.027; *t*-test; Figure 4B]. In contrast, tau phosphorylation in AT8 was increased in CA1 and CA3, while no changes were observed in the dentate gyrus [Ctrl vs. Epi: CA1, 1.735 ± 0.1525 (mean ± SEM), *p* = 0.014; CA3: 2.452 ± 0.3394 (mean ± SEM), *p* = 0.0073; *t*-test; Figure 4B]. Without correction for total-tau levels, only the CA3 subfield showed significant differences in AT8 phosphorylation [*t*-test, 1.850 ± 0.3097 (mean ± SEM), *p* = 0.038)], while CA1 showed no changes (Supplementary Figure S3B).

In summary, while tau phosphorylation remained elevated during epilepsy at the AT8 epitope, total tau expression was reduced in the CA3 and CA1 subfield of the hippocampus.

### Localization of AT8 Positive Phospho-Tau During Epilepsy

**Figure 5:**
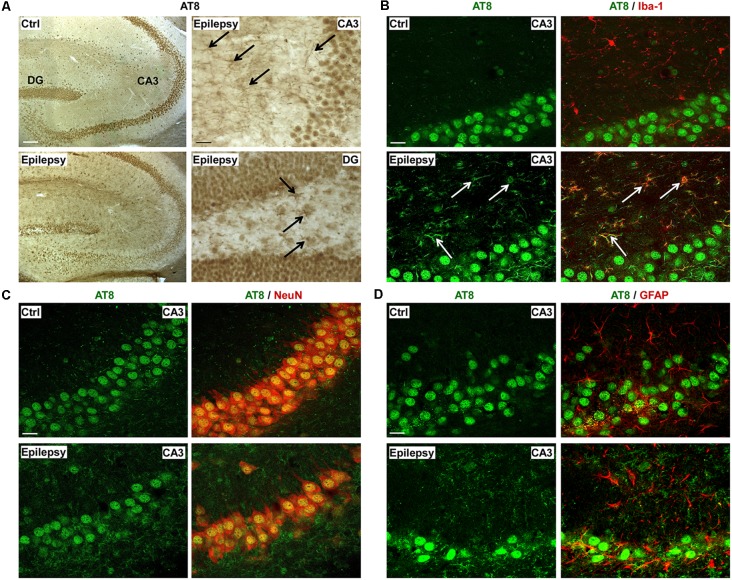
Microglial localization of phospho-tau in the hippocampus of epileptic mice. **(A)** Representative photomicrographs of DAB staining (from a total of three different mice) for AT8 phospho-tau in control and epileptic mice showing the presence of AT8-phospho-tau throughout the entire hippocampus, with an apparent glial-shaped appearance. AT8-positive cells are indicated by black arrows. Scale bar = 200 μm. **(B)** Representative photomicrographs (from a total of three different mice) showing co-localization of AT8 phospho-tau with Iba1-positive microglia marker in the CA3 subfield (indicated by white arrows) during chronic epilepsy. Scale bar = 25 μm. **(C)** NeuN co-staining with AT8 phospho-tau in neurons from the CA3 subfield in epileptic mice. Scale bar = 25 μm. **(D)** Lack of co-localization between astrocyte marker GFAP and AT8 phospho-tau. Scale bar = 25 μm.

To determine in what cell types in the hippocampus phosphorylated tau was localized, immunostaining was carried out as before using the AT8 antibody, which showed the greatest change during epilepsy. In contrast to our results from post-status epilepticus tissue sections, AT8 phospho-tau localization to the mossy fibers was absent in the hippocampus of epileptic mice (Figure 5A). AT8-positive staining could, however, be observed throughout the entire hippocampus including all hippocampal subfields with an apparent glial-shaped appearance (Figure 5A). To establish which cell types express AT8-positive tau in the hippocampus, double immunofluorescence staining was carried out using the cell type-specific markers Iba-1 for microglia, GFAP for astrocytes and NeuN for neurons. This revealed that, AT8, as shown *via* DAB staining, was not detected in the mossy fibers (Figure 5B). While AT8-positive tau strongly co-localized with the microglia marker Iba-1 in all hippocampal subfields (Figure 5C, Supplementary Figures S4A–C), no co-localization was observed between AT8 and the astrocyte marker GFAP (Figure 5D, Supplementary Figures S4A–C).

Taken together, our results show that tau alterations persist within the epileptic state, however, suggest that the pattern of abnormal tau phosphorylation during epilepsy is distinct from status epilepticus.

## Discussion

In the present study, we confirm and further elaborate on the effects of status epilepticus and epilepsy on the phosphorylation of tau in the hippocampus. We identify significant differences in the pattern of tau phosphorylation on specific epitopes between status epilepticus and chronic epilepsy with distinct localization of phosphorylated tau in epileptic mice compared to post-status epilepticus. The identification of a pattern of tau pathology in epilepsy distinct from status epilepticus could aid in elucidating the pathways by which seizure activity may drive neurodegeneration in epilepsies (Tai et al., 2016).

The phosphorylation of tau post-seizure activity has previously been described in models of intraperitoneal delivered KA-induced status epilepticus with the results of these studies being largely reproduced in our study in addition to the identification of a distinct tau phosphorylation pattern during epilepsy (Crespo-Biel et al., 2007; Liang et al., 2009). These mechanisms of tau phosphorylation have been given little attention as regards to epileptic disorders but could be central to explaining the high incidence of Alzheimer’s disease in epilepsy patients (Téllez-Zenteno et al., 2005).

In our model, we detected an increase in protein levels of tau, which was not previously observed in other KA seizure models. Notably, the increase in tau protein levels did not correspond to any significant change in* tau* mRNA, indicating that the observed increase is not due to an increase in transcription and with no increase in total-tau present at 14 days it is likely a short-lived effect. Why tau was increased in the dentate gyrus and decreased in CA3 we do not know. A possible explanation is the inhibition of the ubiquitin-proteasome system (UPS) following status epilepticus. We have previously shown status epilepticus-induced inhibition of the UPS mainly to occur in the dentate gyrus, while proteasome activity has been shown to be slightly increased in the CA3 subfield of the hippocampus post-status epilepticus (Engel et al., 2017).

The subfield-specific changes in tau phosphorylation are possibly a result of the subfield-specific difference in kinase/phosphatase activity which may be dynamically altered in response to seizure activity which has similarly been described in other models (Liang et al., 2009). In line with this, we have previously shown that there are subfield-specific differences in GSK-3β activity following intraamygdala KA-induced status epilepticus (Engel et al., 2018). The reason for the observed early increase followed by a strong decrease in tau phosphorylation in CA3 post-status epilepticus remains elusive. The cell death-prone CA3 subfield seems to be, however, particularly susceptible to seizures in the intra-amygdala KA mouse model potentially leading to the activation of distinct intracellular signaling cascades during seizures when compared to the from cell death protected CA1 and dentate gyrus. Interestingly, previous studies have shown that the enhancement of tau phosphatase PP2A activity through the treatment with the PP2A activator sodium selenate to be an effective anti-epileptic treatment which further illustrates the dynamic relationship of tau and epileptiform activity and could present a novel therapeutic avenue for potentially protecting against seizures and the development of tau pathology (Jones et al., 2012). It must also be acknowledged that epilepsy and seizure activity have been repeatedly shown to have dramatic effects on post-transcriptional regulation *via* microRNAs, which could be implicated in the changes in tau expression observed in this study with various microRNAs altered in epilepsy models also known to target tau (Jimenez-Mateos and Henshall, 2013; Santa-Maria et al., 2015; Zheng et al., 2016). Cell death in the CA3 and hilus that occurs post-status epilepticus may also affect the levels of phospho-tau reducing the tau-containing cells and thus AT8-phosphorylated tau (Fuster-Matanzo et al., 2012). Status epilepticus stimulates sprouting of mossy fibers from the granule cell layer and interestingly, we observed AT8 phospho-tau co-localizing to the mossy fibers possibly contributing to sprouting observed during epileptogenesis (Parent et al., 1997).

Tau phosphorylation at 1–24 h time-points post-status epilepticus was most prominent on serine/threonine 202/205 (AT8) compared to serine 396/404 (PHF-1) with only AT8 phospho-tau increased in epileptic mice at 14 days. AT8 phosphorylation is the standard immunostaining utilized for the assessment of neurofibrillary tangle (NFT) formation and tau aggregation for Braak stage scoring and Alzheimer’s Disease progressions, with a very strong correlation with cognitive decline in preclinical Alzheimer’s Disease (Braak et al., 2006; Huber et al., 2018). The phosphorylation at Ser202/Thr205 (AT8) is associated with pre-tangle formation stage and among the earliest sites to be aberrantly phosphorylated in Alzheimer’s Disease and is known to induce a toxic gain-of-function (Su et al., 1994; Luna-Muñoz et al., 2007; Kanaan et al., 2011). AT8 phospho-tau aggregates were also identified in the hippocampus and cortex of TLE patients and correlated with cognitive decline (Tai et al., 2016). The lack of phosphorylation in PHF-1 (Ser-396/404) compared to AT8 (Ser202/Thr205) epitopes is interesting with regard to the activity of specific kinases on these sites as while GSK-3β and CDK5 both phosphorylate these epitopes, CDK5 acts on Ser-202 to a far greater extent with GSK-3β more prominently targeting Ser-396 (Cavallini et al., 2013). We had previously investigated the importance of GSK-3β during seizures through inhibition with NP12 and when investigated with regard to its effect on tau phosphorylation the lack of any significant effect in ameliorating the hyperphosphorylation of tau post-status suggests that the phosphorylation of tau occurs independently of GSK-3β. In addition, the lack of PHF-1 phosphorylation at 24 h and at 14 days, while AT8 phosphorylation remains elevated, could indicate that CDK5 activity has a more influential effect on the continued phosphorylation of tau in epileptic mice.

In contrast to status epilepticus, during epilepsy tau expression was not increased in the dentate gyrus, but reduced in the hippocampal subfields CA3 and CA1. Again, it is tempting to speculate that this is likely a protective response to potentially toxic levels of phosphorylated tau in the cells occurring during seizures. Another novel observation during the study was the presence of phosphorylated tau in microglia during chronic epilepsy. Tau is not natively expressed in microglia and its presence is most likely due to active microglia internalizing extracellular tau as has been previously described *in vitro* and *in vivo* using transgenic models of tauopathy (Luo et al., 2015; Bolós et al., 2016). Internalization of extracellular tau by microglia is thought to be protective, accelerating the clearance of toxic forms of tau in diseased brains although the activation of microglia is implicated in several pathways of epileptogenesis as well as driving the spread of tau pathology (Maphis et al., 2015). Extracellular tau is typically attributed to cell death but with no obvious cell death occurring during chronic epilepsy in our model, this is unlikely to be the primary contributor (Moran et al., 2013). One mechanism of extracellular tau release which could have a significant influence on tau present in epileptic mice is the neuronal excitation stimulated tau release described by Pooler et al. (2013) which could potentially result in a large release of tau through the aberrant excitation in the model. It must be noted that this mechanism has never been evaluated in response to seizures or experimental epilepsy and would require further investigation to confirm but could reveal an important mechanism in the development of tau pathology in epilepsies. These changes in tau phosphorylation in epileptic mice observed in this study have not been previously reported, which is possibly due to previous investigations being limited to status epilepticus without a confirmed epileptic state. To fully elucidate the impact of tau phosphorylation in epileptic mice further studies with longer periods of experimental epilepsy in mice would likely be required as even within tau overexpressing mouse models, tau pathology seeding takes over 1 month to onset (Holmes et al., 2014).

The aberrant tau hyperphosphorylation occurring in epileptic mice presents a convergence of numerous mechanisms that could have significant implications with epilepsies as well as Alzheimer’s Disease. These findings point to experimental epilepsy models examined over a longer period of time to replicate the potential mechanisms of tau hyperphosphorylation and aggregation which have been observed in TLE patients. The intra-amygdala KA mouse model of status epilepticus represents, therefore, a valid model that could be utilized to further elucidate developing treatments against the propagation of hyperphosphorylated tau occurring in TLE patients.

## Data Availability Statement

All datasets generated for this study are included in the article/Supplementary Material.

## Ethics Statement

The animal study was reviewed and approved by Research Ethics Committee of the Royal College of Surgeons in Ireland.

## Author Contributions

MA carried out Western blotting, PCRs, immunostainings and analyzed the data. AK analyzed the data and wrote parts of the manuscript. GL performed immunostainings. EB carried out experiments with GSK-3 inhibitors. TE wrote the manuscript.

## Conflict of Interest

The authors declare that the research was conducted in the absence of any commercial or financial relationships that could be construed as a potential conflict of interest.

## Footnotes

^1^http://frodo.wi.mit.edu
