# Supplementary material for: Tau Phosphorylation in a Mouse Model of Temporal Lobe Epilepsy
Source: Front Aging Neurosci. 2019 Nov 12;11:308. doi: 10.3389/fnagi.2019.00308 (PMC6861366; doi:10.3389/fnagi.2019.00308)
Supplement: Supplementary file 1 [file Table_1.docx]

**Supplementary File**

**Supplementary Figure S1.** *Increased tau phosphorylation and expression in the hippocampus following status epilepticus.*

**
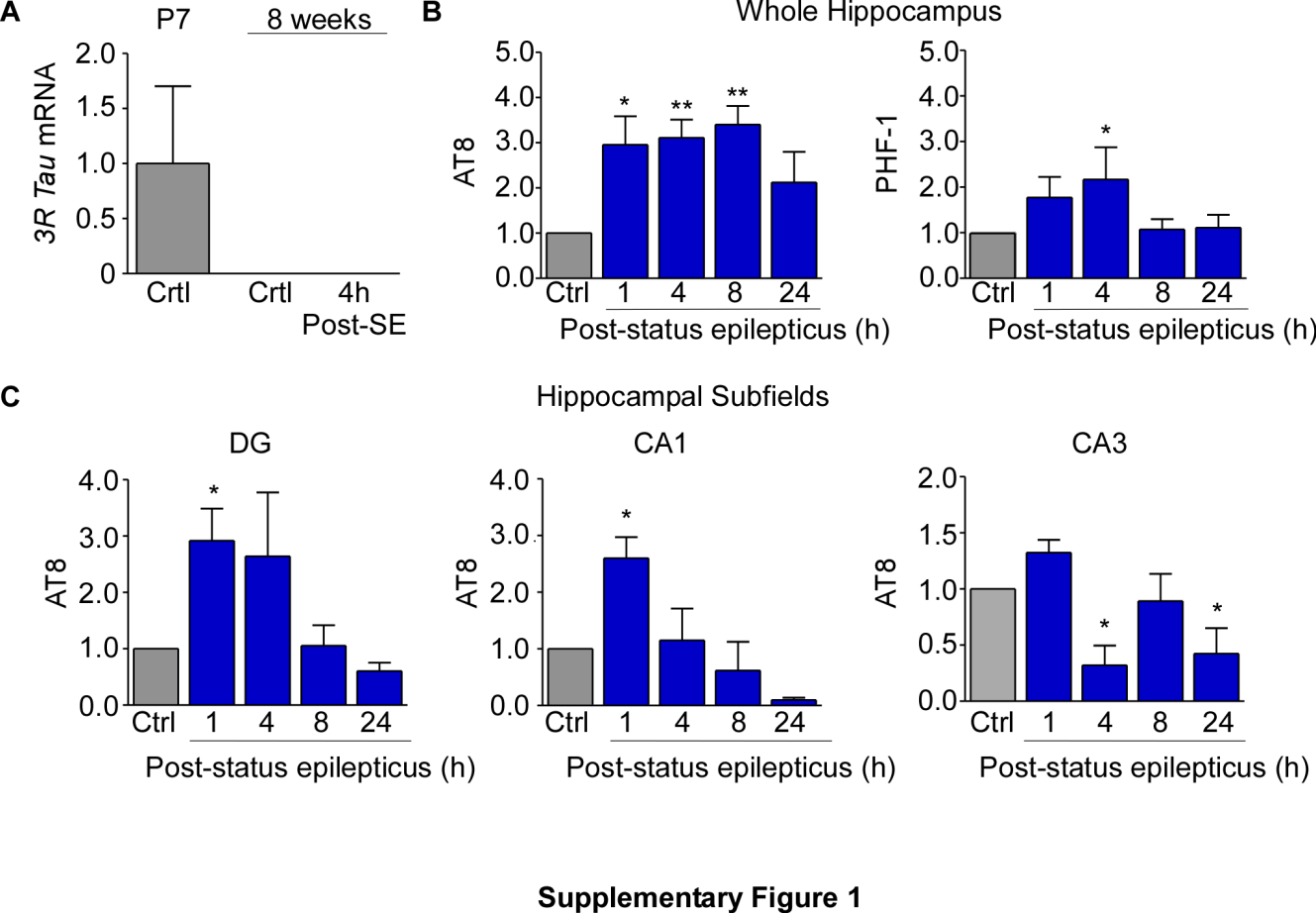
**

**(A)** No amplification of *3R Tau* isoform mRNA in the hippocampus of control mice and 4 h post-status epilepticus (mice were 8 weeks of age). Hippocampal tissue from mice killed at postnatal day 7 (P7) was used as positive control (n = 3 / group). **(B)** Representative Western blots (n = 1 per lane) and corresponding graphs showing increased AT8 and PHF-1-positive tau post-status epilepticus in whole hippocampus when corrected to the loading control GAPDH (AT8: Ctrl vs 1 h post-status epilepticus, p = 0.01; Ctrl vs 4 h post-status epilepticus: p = 0.008; Ctrl vs 8 h: p = 0.003; PHF-1: Ctrl vs 4 h: p = 0.04) (n = 4 per group, one-way ANOVA with Fisher’s post hoc test). **(C)** Representative Western blots (n = 1 per lane) and corresponding graphs of AT8 protein levels in the different hippocampal subfields. AT8 is up-regulated at 1 h post-status epilepticus in the dentate gyrus: Ctrl vs 1 h post-SE: p = 0.046) and CA1 (Ctrl vs 1 h post-status epilepticus: p = 0.013), and down-regulated at 4 h (p = 0.016 and 24 h (p = 0.033) post-status epilepticus in the CA3. (n = 3 per group, one-way ANOVA with Fisher’s post hoc test). *p < 0.05, **p < 0.01. Data are present as mean ± SEM.

**Supplementary Figure S2.** *No effect of the inhibition of GSK-3 on the level of phosphorylation on the AT8 and PHF-1 epitope in the hippocampus.*


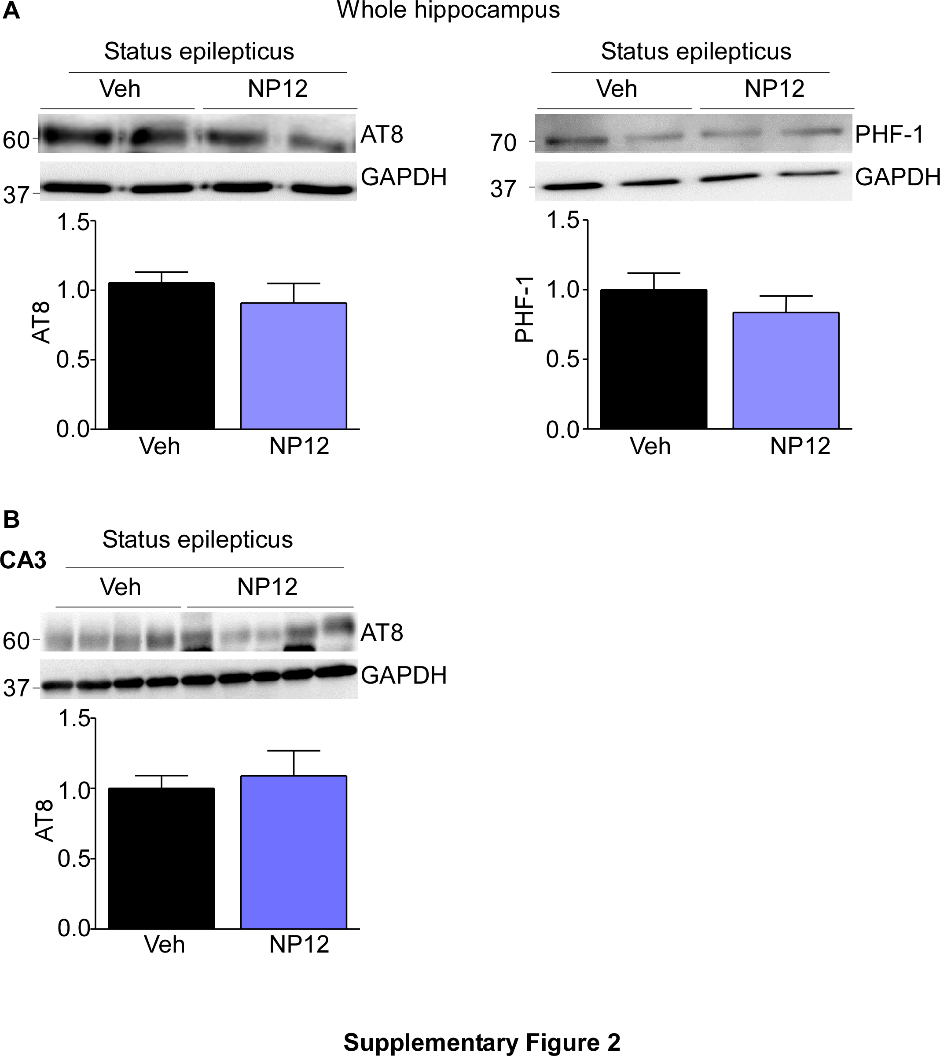


**(A)** Representative Western blots (n = 1 per lane) and corresponding graphs showing similar levels of AT8-dependent and PHF-1-dependent tau phosphorylation post-status epilepticus in the whole hippocampus of mice treated with the GSK-3 inhibitor NP12 4 h following status epilepticus (n = 8 per group, unpaired Student's t test). **(B)** Representative Western blots (n = 1 per lane) and corresponding graphs showing no difference in AT8 phosphorylation in the CA3 subfield of the hippocampus between vehicle-treated and NP12-treated mice 4 h post-status epilepticus (n = 4 vehicle (Veh) and 5 (NP12), unpaired Student's t test). Data are present as mean ± SEM.

**Supplementary Figure S3.** *Subfield-specific tau phosphorylation post-status epilepticus.*

**
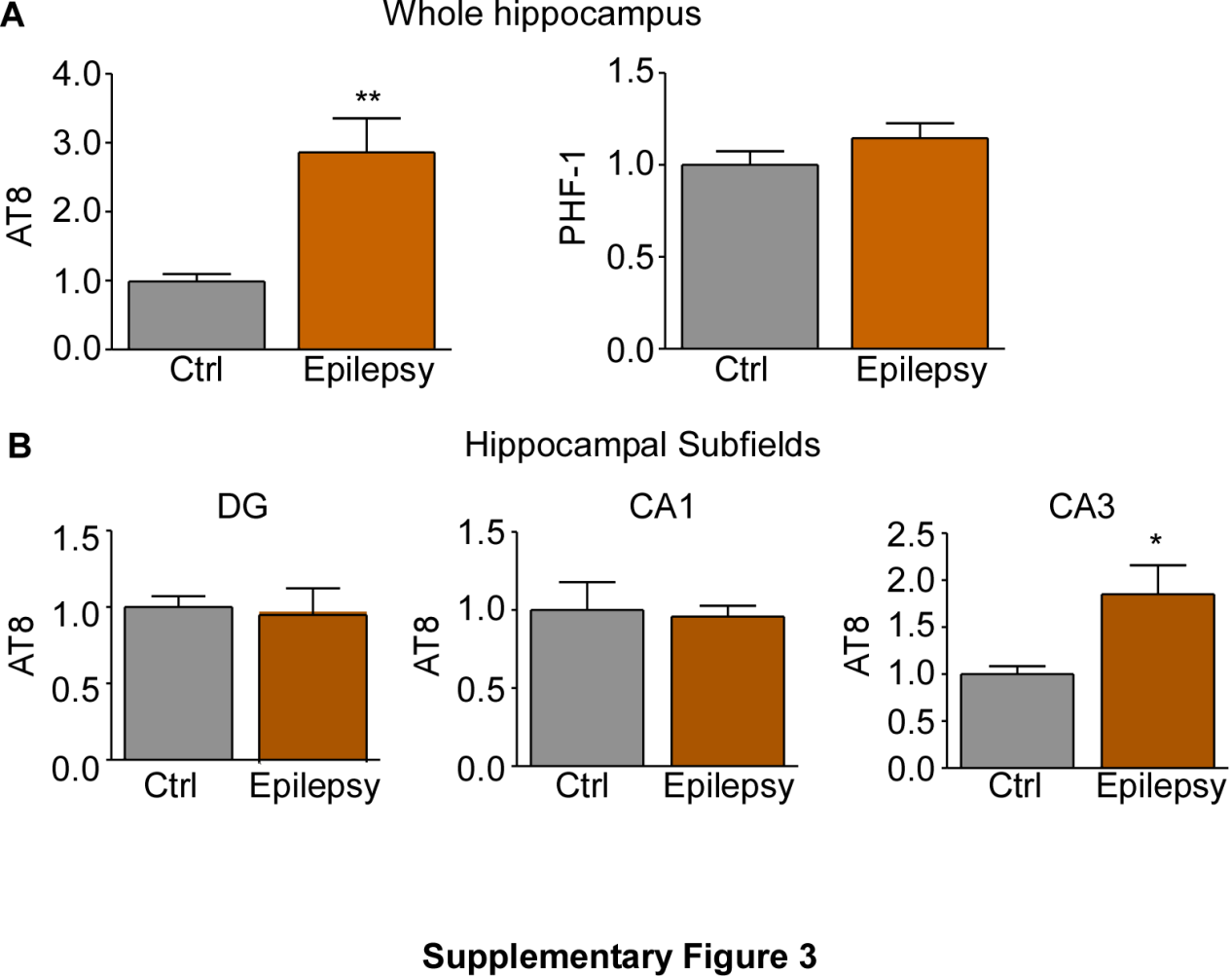
**

**(A)** Representative Western blots (n = 1 per lane) and corresponding graphs showing increased AT8 phosphorylated tau in epileptic mice (Ctrl vs epilepsy: p = 0.002, unpaired Student's t test). No increase was observed at the PHF-1 epitope (n = 10 per group, unpaired Student's t test). **(B)** Representative Western blots (n = 1 per lane) and corresponding graphs showing increased AT8 phosphorylation in the CA3 subfield of the hippocampus in epileptic mice (Ctrl vs epilepsy: p = 0.08, unpaired Student's t test) (CA1: n = 4 (Ctrl) and n = 6 (epilepsy); CA3 and DG: n = 5 (Ctrl) and n = 6 (epilepsy). Data are present as mean ± SEM. *p < 0.5, **p < 0.01

**Supplementary Figure S4.** *Microglial localization of AT8 phospho-tau in the hippocampus during epilepsy in mice.*


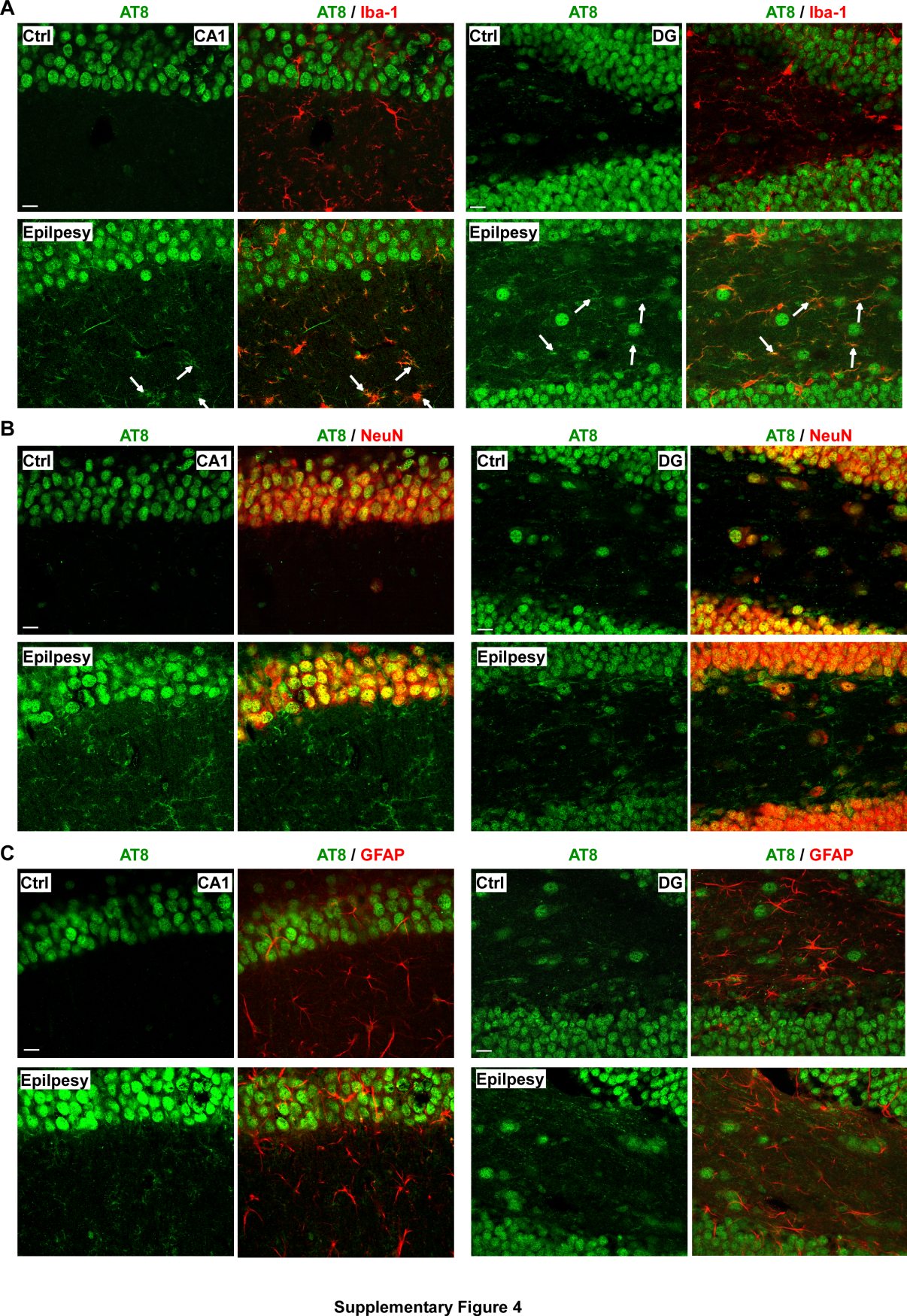


**(A, B)** Representative photomicrograph (out of three different mice) showing co-localisation of AT8 and Iba1-positive microglia in CA1 and dentate gyrus in epileptic mice (indicated by white arrows). Scale bar = 25μm. **(C)** Absence of co-localization of astrocyte marker GFAP with AT8. Scale bar = 25μm.
